# Measurement of M^2^-Curve for Asymmetric Beams by Self-Referencing Interferometer Wavefront Sensor

**DOI:** 10.3390/s16122014

**Published:** 2016-11-29

**Authors:** Yongzhao Du

**Affiliations:** 1 College of Engineering, Huaqiao University, Quanzhou 362021, China; yongzhaodu@hqu.edu.cn; Tel.: +86-188-1599-2396; 2 Fujian Provincial Academic Engineering Research Centre in Industrial Intelligent Techniques and Systems, Huaqiao University, Quanzhou 362021, China

**Keywords:** laser beam characterization, beam quality M^2^-curve, self-referencing interferometer wavefront sensor, fringe pattern analysis

## Abstract

For asymmetric laser beams, the values of beam quality factor $M_{x}^{2}$ and $M_{y}^{2}$ are inconsistent if one selects a different coordinate system or measures beam quality with different experimental conditionals, even when analyzing the same beam. To overcome this non-uniqueness, a new beam quality characterization method named as M^2^-curve is developed. The M^2^-curve not only contains the beam quality factor $M_{x}^{2}$ and $M_{y}^{2}$ in the *x*-direction and *y*-direction, respectively; but also introduces a curve of $M_{x\alpha }^{2}$ versus rotation angle *α* of coordinate axis. Moreover, we also present a real-time measurement method to demonstrate beam propagation factor M^2^-curve with a modified self-referencing Mach-Zehnder interferometer based-wavefront sensor (henceforth SRI-WFS). The feasibility of the proposed method is demonstrated with the theoretical analysis and experiment in multimode beams. The experimental results showed that the proposed measurement method is simple, fast, and a single-shot measurement procedure without movable parts.

## 1. Introduction

Laser beam characterization plays an important role for laser theoretical analysis, designs, and manufacturing, as well as medical treatment, laser welding, and laser cutting and other applications. Therefore, an effective evaluation parameter and its corresponding reliable measurement method for quantifying beam quality are necessary. Among various charactering laser beam quality approaches, the most commonly and widely used approach is the beam propagation factor, simply called ‘beam quality M^2^ factor’ which was first developed by Siegman [1,2] in 1990. The M^2^ factor is defined as the ratio of the beam parameter product of an actual beam to that of an ideal Gaussian beam (TEM_00_) at the same wavelength. That proposal was immediately adopted by the International Organization for Standardization (ISO). The standard draft, ISO/TC172/SC9/WG1, for laser beam characterization and its measurement method, was published in 1991 [3]. Now ISO published the latest version of M^2^ factor measurement Standard ISO11146-1/2/3 [4,5,6], which standardized laser beam characterization by defining all relevant quantities of laser beams including in measurement method instructions. Now M^2^ factor undoubtedly becomes an acceptable characterization parameter for beam quality characterization in the laser community [7,8,9,10,11,12,13,14].

The definition of the beam propagation ratio M^2^ for simple and general astigmatic beams and the instruction for its measurement can be found in the Standard ISO11146 [4,5,6]. The beam quality M^2^ factor is determined by the beam width as a function of propagation distance (*z*) or propagation location of the test beams using hyperbolic fitting approaches. Here, the beam intensity distributions of the test beam measured with a moving CCD camera in various planes is suggested, which allows the determination of the beam widths by using second-order moments of the beam intensity distributions and hence the M^2^ value. However, using this scanning CCD-based method for M^2^ value is quite time-consuming due to the requirements of multiple measurements, consequently, it is unsuitable to characterize the fast dynamics of a laser beam system. In the past decades, some techniques [15,16,17,18,19,20,21,22,23,24] have been developed to measure the dynamic beam quality M^2^ factor, such as the Hartmann wavefront sensor [15,16], but it was shown to yield inaccurate results for multimode beams [17,18]. Other methods to perform M^2^ comply with ISO11146 techniques, for example, multi-plane imaging using distortion diffraction gratings [19], optical field reconstruction using modal decomposition [20,21], and spatial light modulator method [22]. The self-reference interferometer wavefront sensor (SRI-WFS) method [23,24] is another effective method for real-time measurement of M^2^ factor which was developed recently. In general, SRI-WFS is based on a point diffraction interferometer, and its reference wave is generated by the pinhole diffraction with a pinhole filter and it no longer needs an artifacts reference beam, therefore, it is called “self-referencing” [25,26]. The SRI-WFS are usually used as a wavefront diagnosis tool for wavefront phase measurement. However, it cannot measure amplitude (or intensity) at the same time [25,26]. In Reference [24], a modified Mach-Zehnder point diffraction interferometer is developed for reconstructing complex amplitudes including in phase and amplitude (or intensity), and beam quality, M^2^ factor, can be calculated starting from the complex amplitude field of the test beams by using numerical method conforming to the ISO11146 standard [24].

For the asymmetric laser beams such as simple astigmatic beam and complex astigmatic beam [27,28], $M_{x}^{2}$ and $M_{y}^{2}$ are usually used to evaluate the beam quality in the *x*-direction and the *y*-direction, respectively [4,5,6]. However, we found that the values of $M_{x}^{2}$ and $M_{y}^{2}$ may change or are inconsistent if the coordinate axis is rotated. In other words, they are coordinate system dependent and change under different experimental conditions even when analyzing the same beam. Therefore, just using the simple parameters of $M_{x}^{2}$ and $M_{y}^{2}$ to characterize the beam quality of an asymmetric beam, such as a simple astigmatic beam and complex astigmatic beam, are not comprehensive or objective [9,29]. In references [30,31,32], a new beam characterization method, beam quality M^2^-matrix, is developed. It not only contains the beam quality terms, $M_{x}^{2}$ and $M_{y}^{2}$, to evaluate the beam quality in *x*-direction and *y*-direction, but also introduces another cross term, M*_xy_*, which is used to characterize the cross-relationship between x-direction and y-direction [30,31]. The M^2^-matrix has more general physical meaning and wider application scope both for asymmetric beams and astigmatic beam [32]. 

In this paper, a new beam quality characterization method, beam quality M^2^-curve, for an asymmetric laser beam is developed. The M^2^-curve not only contains the beam quality factor $M_{x}^{2}$ and $M_{y}^{2}$ in the *x*-direction and *y*-direction, respectively; but also introduces a curve of $M_{x\alpha }^{2}$ versus rotation angle *α* of coordinate axis. Moreover, we also present a real-time measurement method to demonstrate beam propagation factor M^2^-curve with a modified self-referencing Mach-Zehnder interferometer based-wavefront sensor (henceforth SRI-WFS). By using the SRI-WFS, a full characterization of laser beams both amplitude or intensity profile and wavefront phase are reconstructed from a single fringe pattern with Fourier transform (FT) based spatial phase modulation technology [33]. The beam quality M^2^-curve can be obtained starting from the complex field of the test laser beam by using the virtual caustic method conforming to the ISO standard method [4]. The feasibility of the proposed method is demonstrated with the theoretical analysis and experiment in asymmetric (Hermite-Gaussian-like mode) beams. The article is arranged as follows: The first section is the introduction. The theoretical analysis and derivation of SRI-WFS based complex amplitude reconstruction method is presented in Section 2. According to the Standard ISO11146, the formula derivation process for determining beam quality M^2^-curve starting from the reconstructed complex amplitude is showed in Section 3. In Section 4, the experimental results of beam quality M^2^-curve with the proposed SRI-WFS method are demonstrated. Finally, the conclusions are derived from this work.

## 2. Self-Referencing Interferometer Wavefront Sensor

In this section, a new complex amplitude reconstruction method based on self-referencing Mach-Zehnder interferometer wavefront sensor (SRI-WFS) is presented. Firstly, the SRI-WFS experiment configuration is introduced. Secondly, we give the basic theory of complex amplitude method based on SRI-WFS, including in the theoretical derivation process and fringe pattern analysis method for reconstructing the complex amplitude of the test beam.

### 2.1. Experiment Setup of SRI-WFS

**Figure 1:**
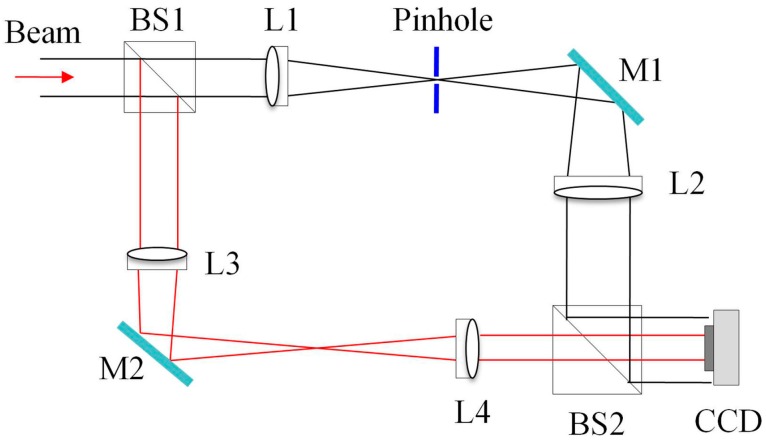
Experimental setup of SRI-WFS: BS1, BS2, beam-splitters, L1–L4, lens with the focal length *f*_1_ = *f*_4_ = 100 mm and *f*_2_ = *f*_3_ = 300 mm, respectively; pinhole, spatial filtering plate with diameter *d_pin_* = 25 μm; M1, M2, reflection mirrors.

The experimental setup of SRI-WFS, which can be seen as a modified Mach-Zehnder radial shearing interferometer [34,35,36], is shown in Figure 1. This optical system consists of two beam-splitters, BS1 and BS2; a spatial filter, pinhole; two mirrors, M1 and M2; a two-lens positive telescope imaging systems which consisted of two lens, L1 and L2, with focal lengths of *f*_1_ = 100 mm and *f*_2_ = 300 mm, respectively; and another two-lens positive telescope imaging systems which consisted of two lens, L3 and L4, with focal lengths of *f*_3_ = 300 mm and *f*_4_ = 100 mm, respectively. A pinhole plate, functioning as a low-pass spatial filter, is placed at the common focal plane of Lens L1 and L2. The signal and reference waves formed interference pattern in the CCD plane. Here we define, g = *f*_2_/*f*_1_ = *f*_3_/*f*_4_, and the magnification of SRI-WFS is defined as *G* = *g*^2^. Compared to the tradition radial shearing interferometer configuration, in our experimental setup as shown in Figure 1, we made an improvement by simultaneously using a pinhole filter and a telescope system in each arm of SRI-WFS to generate a promising reference wave.

### 2.2. Reconstruction of Complex Amplitude Field

For simplicity, we denote the field of the incident test beam as,

(1) E(r,φ)=u(r,φ)exp[iϕ(r,φ)],

where *r* and *ϕ* are radial and circular coordinates, respectively; *u*(*r*, *ϕ*) denotes the amplitude and *φ*(*r*, *ϕ*) is the phase information of incident test beam. As shown in Figure 1, the incident beam was split into two beams by the beam-splitter BS1, the reflected beam, and transmitted beam, which acted as signal wave and reference wave, respectively. The reflected beam travels along the invert telescope which consists of lens L3 and L4, and forms a constructed beam and served as a signal wave,

(2) $$E_{s}\left(gr,\varphi \right)=g\sqrt{R}\cdot u\left(gr,\varphi \right)\exp\left[i\phi \left(gr,\varphi \right)\right]$$

Here, *g* is the amplification factor of the invert telescope and it is defined as *g* = *f*_3_/*f*_4_; *R* in the reflectivity of BS1. The signal wave contains all the information (including in the amplitude *u*(*gr*, *ϕ*) and the phase *φ*(*gr*, *ϕ*)) of the tested beam *E*(*r*, *ϕ*). The transmitted beam served as a reference wave which can be deduced by Fourier optics theory [37,38,39,40],

(3) $$E_{r}\left(\frac{r}{g},\varphi \right)=\frac{\sqrt{1-R}}{g}\cdot u\left(\frac{r}{g},\varphi \right)\exp\left[i\phi \left(\frac{r}{g},\varphi \right)\right]⊗T\left(\frac{r}{g},\varphi \right),$$

where *u*(*r/g*, *ϕ*) represents the amplitude of the reference wave; and (*r/g*, *ϕ*) is the wavefront phase of the reference wave; ⊗ is the two-dimensional convolution operator. *T*(*r*/*g*, *ϕ*) is the Fourier transform of the pinhole with diameter *d_pin_*, and it is given by [40],

(4) $$T\left(\frac{r}{g},\varphi \right)=\frac{\pi d_{pin}^{2}}{4\lambda f_{1}}\cdot 2J_{1}\left[\frac{\pi d_{pin}r}{s\cdot \lambda f_{1}}\right]/\left[\frac{\pi d_{pin}r}{s\cdot \lambda f_{1}}\right].$$

Here *J*_1_ is a first-order Bessel function of the first kind. 

The signal wave *E_s_*(*gr*, *ϕ*) and the reference wave *E_r_*(*r/g*, *ϕ*) interfere in their superposition area (*gr*, *ϕ*). Therefore, the fringe pattern produced by the signal wave *E_s_*(*gr*, *ϕ*) and the reference wave *E_r_*(*r/g*, *ϕ*) captured by the CCD can be expressed as,

(5) $$I\left(gr,\varphi \right)={\left|E_{r}\left(\frac{r}{g},\varphi \right)\exp\left[i\kappa \left(gr,\varphi \right)\right]+E_{s}\left(gr,\varphi \right)\right|}^{2},$$

where *κ* denotes the linear carrier-frequency coming from an angle between the reference and signal waves, which is formed by slightly tilting beam-splitter BS2.

Considering the contracted signal wave and enlarged reference wave in the SRI-WFS system, we redefine the overlapping area, (*rg*, *ϕ*), responding to the field of the signal wave, as a new coordinate domain (*r′*, *ϕ*). The field of signal wave can be shown as (*r′*, *ϕ*), and the reference wave is shown as *E*(*r′/g*^2^, *ϕ*). Therefore, the Equation (5) can be rewritten as,

(6) $$I\left(r^{'},\varphi \right)={\left|E_{r}\left(\frac{r^{'}}{G},\varphi \right)\exp\left[i\kappa \left(r^{'},\varphi \right)\right]+E_{s}\left(r^{'},\varphi \right)\right|}^{2},$$

where *G* = *g*^2^ is the magnification of the SRI-WFS. For simplicity, we define the overlapping area (*x*/*G*, *y*/*G*) as new coordinate domain (*x*, *y*) and its intensity distribution in the CCD plane can be written into a general form of the fringe pattern, as follows,

(7) $$i\left(x,y\right)\propto a\left(x,y\right)+b\left(x,y\right)\cos\left[2\pi \left(\kappa_{x}x+\kappa_{y}y\right)+\phi \left(x,y\right)\right],$$

where *a*(*x*, *y*) ∝ *E_s_^2^*(*x*, *y*) + *E_r_^2^*(*x*/*G*, *y*/*G*) is the background intensity, and *b*(*x*, *y*) ∝ *2u_s_*(*x*, *y*)*u_r_*(*x/G*, *y*/*G*) is the modulation intensity of fringe pattern; *κ_x_* and *κ_y_* are the carrier-frequency components along *x*-direction and *y*-direction, respectively. Therefore, the complex amplitude of modulation (CAM) function [41] of the interferogram as shown in Equation (7) can be easily extracted by spatial phase modulation (SPM) technology, proposed firstly by M. Takeda [33]. The CAM function contains both information of the wavefront phase *φ*(*x*, *y*) and amplitude *E_s_*(*x*, *y*) of the test beams.

Taking the Fourier transform in two-dimension for the interference pattern [33,42,43],

(8) $$\begin{array}{cc} I\left(X,Y\right) & =A\left(X,Y\right)+[\mathrm{FT}\left\{\exp\left[-i\phi \left(x,y\right)\right]\right\}⊗B\left(X,Y\right)]⊗\delta \left(X-\kappa_{x},Y-\kappa_{y}\right)\\ & \;+\left[\mathrm{FT}\left\{\exp\left[i\phi \left(x,y\right)\right]\right\}⊗B\left(X,Y\right)\right]⊗\delta \left(X+\kappa_{x},Y+\kappa_{y}\right), \end{array}$$

where ***FT***{ } denotes the Fourier transform, *X* and *Y* are the spatial frequency variables, and *δ*(*X*, *Y*) is the Dirac’s delta function, A(*X*, *Y*) and B(*X*, *Y*) presents the frequency-spectrum of a(*x*, *y*) and b(*x*, *y*), respectively. The second and third term in Equation (8) are two Dirac’s functions placed in (*κ_x_*, *κ_y_*) and (*κ_x_*, *κ_y_*) around the low spatial frequency *A*(*X*, *Y*), respectively. By using of a band pass filter, it is possible to extracts the Dirac delta function *δ*(*X* − *κ_x_*, *Y* − *κ_y_*). Then, tacking the inverse two-dimension Fourier transform of the Dirac’s delta function *δ*(*X* − *κ_x_*, *Y* − *κ_y_*), that is, obtaining the CAM function, as follows [24],

(9) $$c\left(x,y\right)=\frac{1}{2}b\left(x,y\right)\propto u_{s}\left(x,y\right)u_{r}\left(\frac{x}{G},\frac{y}{G}\right)\exp\left[-i\phi \left(x,y\right)\right].$$

In this equation, it can be seen that it is similar to the description of complex amplitude of the test wave which is expressed in Equation (1). If the magnification *G* of SRI-WFS is large enough, the term *E_r_*(*x*/*G*, *y*/*G*) will approach to a uniform plane wave, that is, the amplitude tends to *u_r_*(0, 0) and the phase component tends to an ideal plane *φ*(0, 0). Taking into account the amplitude is a relative value in practical application, and the coefficient *u_r_*(0, 0) in the Equation (9) can be rewritten as a constant C. Thus, the complex amplitude distribution of test laser beam can be expressed as [24],

(10) $$E\left(x,y\right)\propto \underset{G\to \infty }{\lim}c\left(x,y\right)=C\cdot u_{s}\left(x,y\right)\exp\left[-i\phi \left(x,y\right)\right].$$

## 3. Determination of M^2^-Curve

### 3.1. Measurement Method for Beam Quality Factor M^2^ Based on the Complex Amplitude Distribution [24]

Once the complex amplitude field *E*(*x*, *y*, 0) of the test laser beam is obtained, according to the diffraction integral theory [37,44], the complete complex amplitude fields *E*(*x*, *y*, *z*) at *z* planes after propagation distance *z* can be calculated using a double fast Fourier transform algorithm [44],

(11) $$E\left(x,y,z\right)=FT^{-1}\left\{\mathrm{FT}\left\{E\left(x,y,0\right)\right\}\exp\left\{\mathrm{ikz}\left[1-\frac{\lambda^{2}}{2}\left(\eta_{x}^{2}+\eta_{y}^{2}\right)\right]\right\}\right\},$$

where *z* is the propagation distance, k is wave number, *λ* is wavelength of test laser beam, *η_x_* = *x*/*λ_z_*, *η_y_* = *y*/*λ_z_* are the spatial frequencies, respectively. The direct (***FT***) and inverse (***FT***^−1^) Fourier transform are calculated using a Fast Fourier Transform algorithm. Furthermore, in accordance with the ISO standard [4,5,6], the complex amplitude fields *E*(*x*, *y*, *z*) can be used to determine the beam parameters relevant for beam propagation, i.e., beam widths *w_x_*(*z*) and *w_y_*(*z*). Performing a hyperbolic fit to the beam width along the beam transmission axis,

(12) $$w_{x,y}^{2}(z)=a_{x,y}z^{2}+b_{x,y}z+c_{x,y},$$

where *w_x_*_,*y*_ is the beam width in the *x*-direction or *y*-direction; *a_x,y_*, *b_x,y_* and *c_x,y_* are the hyperbolic fitting coefficients, and the subscript *x* and *y* correspond to the values along the *x*-direction or *y*-direction, respectively. Then the waist radius *w_x_*_0,*y*0_, divergence half angle *θ_x,y_* and beam propagation factor $M_{x,y}^{2}$ can be calculated from the following expressions [4],

(13) $$w_{x0,y0}^{2}=c_{x,y}-\frac{b_{x,y}^{2}}{4a_{x,y}};$$

(14) $$\theta_{x0,y0}^{2}=a;$$

(15) $$M_{x,y}^{2}=\frac{\pi }{\lambda }\cdot \sqrt{a_{x,y}c_{x,y}-\frac{b_{x,y}^{2}}{4}}.$$

According to above analysis (Section 2.1, Section 2.2 and Section 3.1), as long as the phase *φ*(*x*, *y*) and the amplitude *u*(*x*, *y*) of the test laser beam are obtained, it is possible to calculate the beam quality M^2^ factor, specifically, the values of $M_{x}^{2}$ and $M_{y}^{2}$ along *x*-direction and *y*-direction of the test beams, respectively.

### 3.2. M^2^-Curve

The output beam of a multimode-laser stable cavity with rectangular geometry in many transverse modes can be written as an incoherent superposition of Hermite-Gaussian (HG) beams [45,46]. Usually the spatial modes are separable; the complex amplitude field distribution of HG modes, at the plane *z* = 0, can be written as [45],

(16) $${\mathrm{HG}}_{mn}\left(x,y,0\right)=\frac{1}{\sqrt{w_{x0}w_{y0}}}\sqrt{\frac{2}{2^{m+n}\pi m!n!}}H_{m}\left[\frac{\sqrt{2}}{w_{x0}}x\right]\exp\left[-\frac{x^{2}}{w_{x0}^{2}}\right]\times H_{n}\left[\frac{\sqrt{2}}{w_{y0}}y\right]\exp\left[-\frac{y^{2}}{w_{y0}^{2}}\right],$$

where the phase term is ignored, $w_{0}$ is the waist radius of the fundamental mode HG_00_; and H*_m_* and H*_n_* denotes the Hermite-polynomial of order *m* in *x*-direction and order *n* in *y*-direction, respectively. HG modes are one set of orthogonal eigenfunctions of the scalar Helmholtz wave equation. Due to the completeness of this eigenfunction set, an arbitrary transverse wave field *E*(*x*, *y*, *z*) can be expanded into a superposition of HG modes [46],

(17) $$E\left(x,y,z\right)=\sum_{m=0}^{\infty }\sum_{n=0}^{\infty }c_{mn}{\mathrm{HG}}_{mn}\left(x,y,z\right),$$

with

(18) $$c_{mn}=\iint_{\infty }{\mathrm{HG}}_{mn}^{*}E\left(x,y,z\right)dxdy,$$

where the asterisk denotes complex conjugation operator; *c_mn_* is the complex-valued expansion coefficients, *c_mn_* = *C_mn_*exp(*φ_mn_*), including in the modal amplitude *C_mn_* = |*c_mn_*| and phase *φ_mn_* = angle(*c_mn_*). 

**Figure 2:**
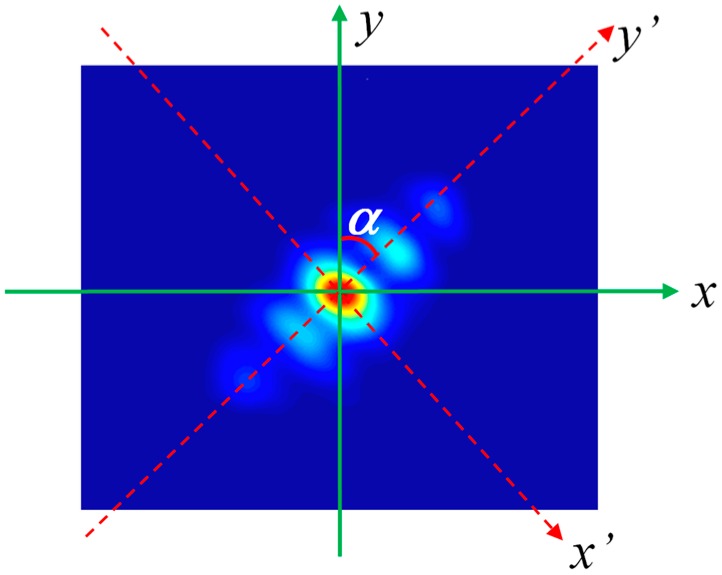
Asymmetric beam intensity distribution in the laboratory coordinate system.

As the analysis above, generally, the intensity profile of the output beam emitted by a multimode stable cavity is non-rotational symmetric or asymmetric beams, as shown in Figure 2. According to Sigman’s theories [1,2], the quality of the laser beam is evaluated by $M_{x}^{2}$ in *x*-direction and $M_{y}^{2}$ in *y*-direction. However, we found that the values of $M_{x}^{2}$ and $M_{y}^{2}$ are non-unique if we rotate coordinate the axis in an astigmatic laser beam. Consequently, just using the simple parameters $M_{x}^{2}$ and $M_{y}^{2}$ to characterize the beam quality of an asymmetric beam is not comprehensive or objective [9,29,30].

Similarly to the traditional definitions form of the beam widths in the general astigmatic beam [4], we have

(19) $$w_{xz}^{2}=\frac{4\int \int_{-\infty }^{+\infty }I(x,y,z){\left(x-\bar{x}\right)}^{2}dxdy}{\int \int_{-\infty }^{+\infty }I(x,y,z)dxdy}$$

and

(20) $$w_{yz}^{2}=\frac{4\int \int_{-\infty }^{+\infty }I(x,y,z){\left(y-\bar{y}\right)}^{2}dxdy}{\int \int_{-\infty }^{+\infty }I(x,y,z)dxdy}$$

and

(21) $$w_{xyz}=\frac{4\int \int_{-\infty }^{+\infty }I(x,y,z)(x-\bar{x})(y-\bar{y})dxdy}{\int \int_{-\infty }^{+\infty }I(x,y,z)dxdy}$$

and

(22) $$w_{rz}^{2}=\frac{4\int \int_{-\infty }^{+\infty }I(x,y,z)\left[{\left(x-\bar{x}\right)}^{2}+{\left(y-\bar{y}\right)}^{2}\right]dxdy}{\int \int_{-\infty }^{+\infty }I(x,y,z)dxdy}$$

where $\bar{x}$ and $\bar{y}$ are the centroid coordinates of the power density distribution which defined as the first-order moment of the power density distribution of the test beams. Therefore, the corresponding beam divergence half angles are given by [4],

(23) $$\theta_{x}=\underset{z\to \infty }{\lim}\frac{w_{xz}}{z}$$

(24) $$\theta_{y}=\underset{z\to \infty }{\lim}\frac{w_{yz}}{z}$$

(25) $$\theta_{xy}=\underset{z\to \infty }{\lim}\frac{w_{xyz}}{z}=\underset{z\to \infty }{\lim}\frac{w_{yxz}}{z}$$

(26) $$\theta_{r}=\underset{z\to \infty }{\lim}\frac{w_{rz}}{z}$$

As shown in Figure 2, assuming the rotation angle of the coordinate axis is *α*, we have

(27) $$x_{\alpha }=x\cos\alpha -y\sin\alpha ,y_{\alpha }=x\sin\alpha +y\cos\alpha$$

Therefore, the beam widths and the beam divergence half angles of the original and rotated beam (with a rotation angle *α*) have the following relationships which are given by the Equations (28)–(31), respectively; as following [30,32],

(28) $$w_{xz\alpha }^{2}=w_{xz}^{2}\cos^{2}\alpha +w_{yz}^{2}\sin^{2}\alpha$$

(29) $$w_{yz\alpha }^{2}=w_{xz}^{2}\sin^{2}\alpha +w_{yz}^{2}\cos^{2}\alpha$$

(30) $$\theta_{xz\alpha }^{2}=\theta_{xz}^{2}\cos^{2}\alpha +\theta_{yz}^{2}\sin^{2}\alpha$$

(31) $$\theta_{yz\alpha }^{2}=\theta_{xz}^{2}\sin^{2}\alpha +\theta_{yz}^{2}\cos^{2}\alpha$$

Utilizing the Equations (28)–(31), it is easy to derive the following expressions,

(32) $$w_{x\alpha }^{2}(z)+w_{y\alpha }^{2}(z)=w_{r}^{2}(z)$$

(33) $$\theta_{x\alpha }^{2}+\theta_{y\alpha }^{2}=\theta_{r}^{2}$$

(34) $$\frac{\pi^{2}}{\lambda^{2}}\left(w_{x\alpha }^{2}+w_{y\alpha }^{2}\right)\left(\theta_{x\alpha }^{2}+\theta_{y\alpha }^{2}\right)=\frac{\pi^{2}}{\lambda^{2}}w_{r}^{2}\theta_{r}^{2}.$$

**Figure 3:**
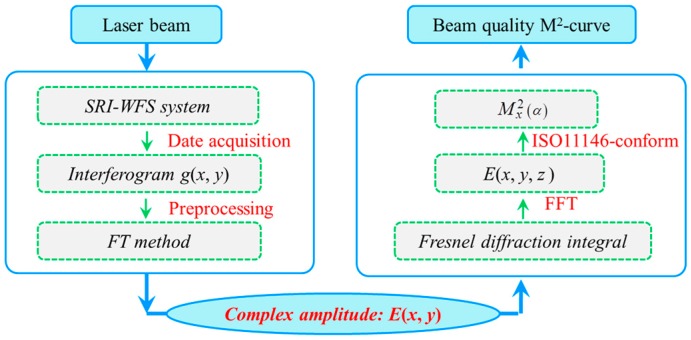
The flow chart of beam quality M^2^-curve determined by the proposed SRI-WFS method.

In this case, we can derive a new beam propagation ratio M^2^(*α*) with rotation angle *α* to characterize the rotated beam and it can be given as,

(35) $$M^{2}(\alpha )=\frac{\pi }{\lambda }w_{x0\alpha }\theta_{x\alpha },$$

where $w_{x0\alpha }$ and $\theta_{x\alpha }$ are waist radius and divergence half angle in the *x*-direction with a rotation angle *α* of coordinate axis, respectively. M^2^ can obtain different values if the rotation angle *α* charges with the coordinate axis, that is, a track of the M^2^ values versus the rotation angle (*α*) of the beam coordinate axis will be form and named M^2^-curve which is unique to a specific tested beam. It can be seen from the Equation (35) that the M^2^-curve takes into account changes in angle and can give a track of the M^2^ values which is compared to the rotation angle *α* of the beam coordinate axis. That is, the M^2^-curve contains all the values of beam quality M^2^ in all directions. The traditional M^2^ factor $M_{x}^{2}$ in *x*-direction and $M_{y}^{2}$ in *y*-direction are just special cases of the M^2^-curve in a special direction of the tested beam, precisely in two orthogonal directions (*x*-axis and *y*-axis). Therefore, the M^2^-curve has a more comprehensive physical meaning and is more objective for characterizing the beam quality because it can be uniquely determined for a particular tested beam. In other words, there is a one-to-one mapping relationship between a particular tested beam and its sharp of M^2^-curve. According to the above analysis in Section 2 and Section 3, we can summarize the flow chart of the M^2^-curve determined by the SRI-WFS method, as shown in Figure 3. 

## 4. Experiment Results and Discussions

The beams from a diode pump solid state laser (DPSSL) with 532 nm wavelength are used as test beam. The excitation of mode mixture in the DPSSL are Hermite-Gaussian-like modes (shorted as TELM_mn_) is achieved by adjusting the tilt angle of the cavity mirrors [45]. The experiment setup of SRI-WFS for completely reconstructing the complex amplitude field of a laser beam is depicted in Figure 1, and it has been demonstrated that it can be used for beam quality measurements in real-time and the details of SRI-WFS system can be seen in [24]. Two invert telescopes with telescope factor *g* = 3, consisted of achromatic lens L1–L4 with focal length *f*_1_ = *f*_4_ = 100 mm and *f*_2_ = *f*_3_ = 300 mm, respecting the magnification *G* = 9 of SRI-WFS. A pinhole plate with diameter *d*_pin_ = 25 μm is used to generate a reference wave. A CCD camera, model MVIC-II-1MM, with 1024 × 1280 pixels and 5.2 micron in each pixel, is positioned at the imaging plane to record interferogram in real time. The CCD sampled-data are sent to the personal computer system (PC) for further processing.

**Figure 4:**
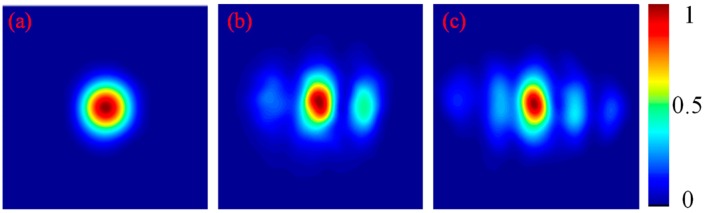
Measured intensity profiles of Hermite-Gaussian-like modes in (**a**) TELM_00_; (**b**) TELM_20_ and (**c**) TELM_40_.

**Figure 5:**
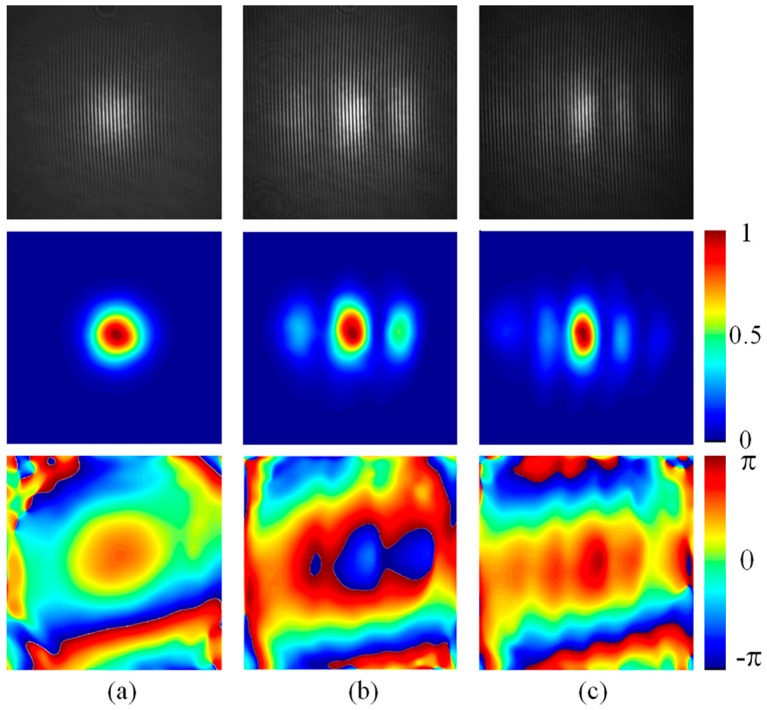
The captured interferogram, reconstructed intensity (a.u.), and wrapped phase maps of Hermite-Gaussian-like modes in (**a**) TELM_00_; (**b**) TELM_20_ and (**c**) TELM_40_. Interferogram (up row), reconstructed intensity profile (middle row), and the reconstructed phase (low row) of the test beams.

Adjusting a slight tilt angle in the cavity mirrors, we can obtain different mix-mode beam outputs whose intensity profiles are similar to the Hermite-Gaussian mode in TEM_mn_. For the convenience of the following analysis, we denote these mix-mode beams as Hermite-Gaussian-like modes in TELM_mn_ to distinguish with the pure Hermite-Gaussian modes in TEM_mn_. As shown in Figure 4, three intensity profiles corresponding to Hermite-Gaussian-like modes in TELM_00_, TELM_20_, and TELM_40_, respectively, are used as the test beams in our experiments. Corresponding interferograms formed with SRI-WFS are shown in Figure 5 (up row). The reconstructed intensity profiles (middle row) and the reconstructed wrapped phase distribution (low row) of the test beams are also shown in Figure 5. Each reconstructed distributions of amplitude and phase for the test beams are extracted from a single interferogram according to Equations (7)–(10). Considering the limited of conditions in our laboratory, we only investigate the intensity distribution for comparative analysis. For a quantitative comparison of the measurement and reconstruction intensity profile, two-dimension cross-correlation coefficient C_c_ is used as a metric [24]. Here C_c_ is defined as the cross-correlation between the images of measurement intensity distribution *I_M_*(*x*, *y*) and reconstruction intensity distribution *I_R_*(*x*, *y*), and C_c_ = 1 is indicated that the measured intensity profile and reconstructed intensity profiles match perfectly. In our experiment, the C_c_ of the reconstructed and measurement intensities for the Hermite-Gaussian-like modes in TELM_00_, TELM_20_ and TELM_40_ are 0.999, 0.998 and 0.997, respectively. 

**Figure 6:**
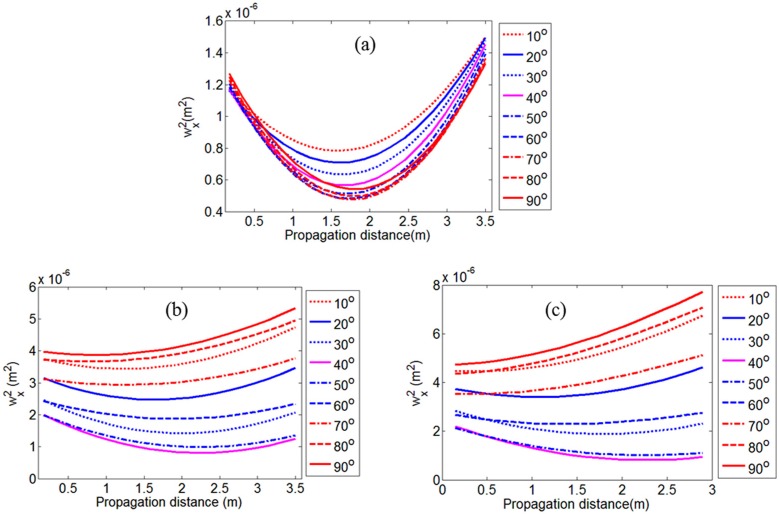
The beam widths *w_x_* in *x*-direction of Hermite-Gaussian-like modes (**a**) TELM_00_; (**b**) TELM_20_; and (**c**) TELM_40_ versus propagation distance. The rotation angle *α* of the coordinate axis is 10, 20, 30, 40, 50, 60, 70, 80, 90°, respectively.

There are several causes for the deviation of the reconstruction intensity and the measured intensity: (1) optical components such as inaccurate errors of telescope system and misalignment in the SRI-WFS system lead to beam distortion; (2) the intensity of the reference wave hardly becomes completely flat with the limited magnification of SRI-WFS in practice; (3) filtering error in the Fourier transform method for interferogram analysis; etc. As long as reconstructing the complex amplitude field *E*(*x*, *y*, 0) of the test beams, the field *E*(*x*, *y*, *z*) at different plane of *z*-axis direction (with different propagation length *z*) can be determined easily according to the Equation (11), and the intensities distribution *I*(*x*, *y*, *z*) = |*E*(*x*, *y*, *z*)|^2^ are also determined uniquely. According to the Standard ISO11146, the beam width *w_xz_* can be determined by the second moment of the obtained intensity distributions, as described in Equations (19)–(22). The beam width *w_x__z_* of test beams in *x*-direction of Hermite-Gaussian-like modes TELM_00_, TELM_20_, and TELM_40_ can be calculated directly from the intensity distribution *I*(*x*, *y*, *z*) at different propagation location *z*. Moreover, according to the Equations (13) and (28)–(31), the beam widths *w_xzα_* and the beam divergence half angles *θ_xzα_* with different rotation angle *α* of the coordinate axis also can be obtained. Figure 6 shows the results of the beam width *w_xzα_* in the *x*-direction of Hermite-Gaussian-like modes (a) TELM_00_; (b) TELM_20_ and (c) TELM_40_ versus propagation distance z and the different rotation angle *α* of coordinate axis. Here, the rotation angles *α* are settled to 10, 20, 30, 40, 50, 60, 70, 80 and 90°; respectively.

**Figure 7:**
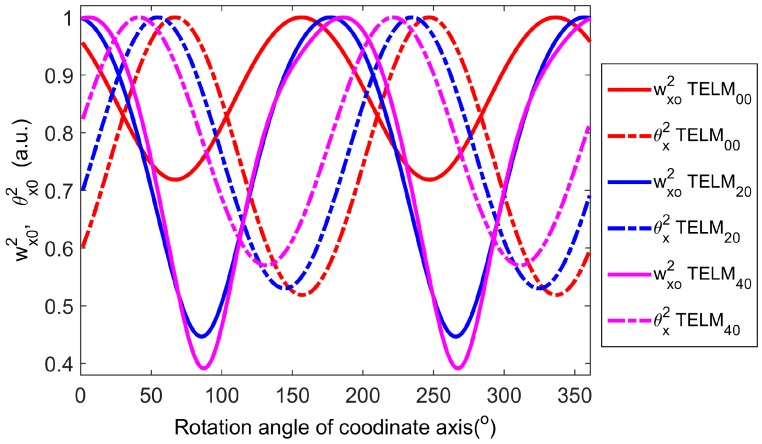
Normalized waist radius w*_x_*_0_ and divergence half angle *θ_x_* in *x*-directions for Hermite-Gaussian-like modes (TELM_00_, TELM_20_, and TELM_40_) versus rotation angle of coordinate axis.

It can be seen from Figure 6 that with the different rotation angle *α*, for example, *α*= 10, 20, 30, 40, 50, 60, 70, 80, and 90°; the beam widths *w_x_*, the beam waist radius *w_x_*_0_, beam divergence half angles *θ_x_*, and waist positions *z*_0_ of the tested beams are all very different. It is further evidenced by the results of the normalized waist radius *w_xα_*(*z*_0_) and beam divergence half angles *θ_x_* in the *x*-directions for Hermite-Gaussian-like modes (TELM_00_, TELM_20_, and TELM_40_) versus rotation angle of coordinate axis *α*, as shown in Figure 7. That is, with the changing rotation angle of the coordinate axis of the tested beam in actual measurement, the waist radius *w_x_*_0_ and divergence half angle *θ_x_* are ever-changes and these different will directly result in the non-unique of the values of M^2^ factor. Obviously, it is consistent with the previous analysis and the motivation of this paper. Moreover, it also indicates that using the simple M^2^ factor, $M_{x}^{2}$ in *x*-direction and $M_{y}^{2}$ in *y*-direction just two special cases (in the two orthogonal directions) of the test beams but missing the most of information in the other random rotation angles *α* of the test beams.

**Figure 8:**
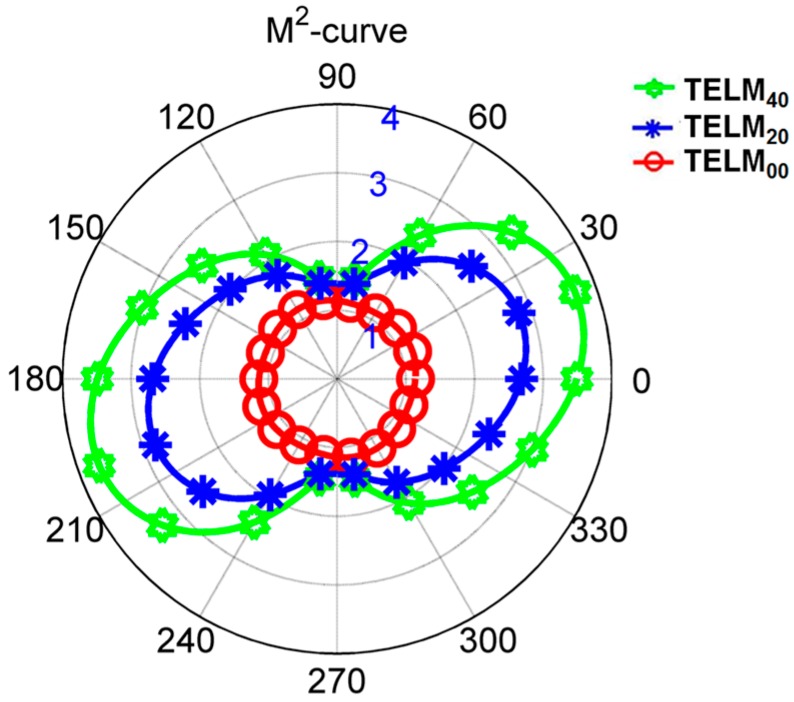
M^2^-curves of Hermite-Gaussian-like modes in TELM_00_, TELM_20_, and TELM_40_. Here, the numbers 1, 2, 3, and 4 are the circular scale for the M^2^-curve.

The results of the M^2^-curves of the test beams (Hermite-Gaussian-like modes TELM_00_, TELM_20_, and TELM_40_) are shown in Figure 8. Note that all the M^2^-curves for Hermite-Gaussian-like modes TELM_00_, TELM_20_, and TELM_40_ are asymmetric. The M^2^-curve is maybe a circle, an ellipse, or an 8-shaped pattern. Moreover, the length values, from the original point to the cross-point at the curve, denote the M^2^ values along this rotation angle *α*. For example, as shown in Figure 8, the values of the test beams, Hermite-Gaussian-like modes TELM_00_, TELM_20_ and TELM_40_ along *α* = 0 direction, which corresponding to the traditional M^2^ factor $M_{x}^{2}$ along *x*-direction of the tested beams, are 1.06, 2.62 and 3.72, respectively. Moreover, when the rotation angle *α* is equal to 90°, corresponding to $M_{y}^{2}$ along the *y*-direction for the tradition M^2^ factor. In this case, the values of $M_{y}^{2}$ are 1.08 for TELM_00_, 1.27 for TELM_20_, and 1.37 for TELM_40_, respectively. For comparative analysis, we also measured the beam propagation factor M^2^ values using the traditional ISO11146 standard based beam quality analyzer (BQA) developed in our previous work [25], and the results are shown in Table 1. It shows that the beam quality M^2^ factors with ISO11146 standard based method are $M_{0x}^{2}$ = 1.04, $M_{2x}^{2}$ = 2.54 and $M_{4x}^{2}$= 3.56 along the *x*-direction and $M_{0y}^{2}$ = 1.06, $M_{2y}^{2}$ = 1.20 and $M_{4y}^{2}$ = 1.32 along the y-direction for Hermite-Gaussian-like modes TELM_00_, TELM_20_, and TELM_40_, respectively. Table 1 also shows the measurement errors between the M^2^ factor values of Hermite-Gaussian-like modes TELM_00_, TELM_20_, and TELM_40_ with the proposed SRI-WFS method and the ISO1116 standard based BQA. The maximum measured deviation of the two methods are 4.49% for $M_{x}^{2}$ and 5.83% for $M_{y}^{2}$ along *x*-axis and *y*-axis, respectively. It can be seen from Table 1 that the results of both measurement methods are well in agreement.

As shown in Figure 8, the M^2^-curve include in all M^2^ information (including in the traditional M^2^ factor values) in all random rotation angle *α* of the coordinate axis. From this sense, M^2^-curve is an extension method for characterization of beam quality. Moreover, its shape will be uniquely determined for a particular test beam. Consequently, it provides a more comprehensive and more objective physical meaning for actual applications. 

## 5. Conclusions

The non-uniqueness of characterization of laser beam quality with the traditional M^2^ factor can be overcome by using the M^2^-curve. The M^2^-curve has more general physical meaning and wider application scope both for asymmetric beams and astigmatic beams. The M^2^-curve not only contains the beam quality terms, $M_{x}^{2}$ and $M_{y}^{2}$, to evaluate the beam propagation quality in the *x*-direction and *y*-direction, respectively; but also introduces a curve of $M_{x\alpha }^{2}$ which is used to characterize beam propagation factor versus the rotation angle *α* of coordinate axis. Moreover, the measurement method of the M^2^-curve based on modified SRI-WFS is also put forward which is used to demonstrate the potential of the proposed method, M^2^-curve, for charactering asymmetric beams. 

## Figures and Tables

**Table 1:** The measurement results of M^2^ factor for Hermite-Gaussian-like modes TELM_00_, TELM_20_, and TELM_40_ with the proposed SRI-WFS method and the ISO1116 standard based beam quality analyzer.

Beam Quality	$M_{x}^{2}$			$M_{y}^{2}$		
	TELM_00_	TELM_20_	TELM_40_	TELM_00_	TELM_20_	TELM_40_
SRI-WFS	1.06	2.62	3.72	1.08	1.27	1.37
ISO1116 method	1.04	2.54	3.56	1.06	1.20	1.32
Errors (%)	1.96%	3.15%	4.49%	1.89%	5.83%	3.79%
